# The impact of dietary supplementation of Quercetagetin on growth, antioxidant capacity, and gut microbiota of diquat-challenged broilers

**DOI:** 10.3389/fmicb.2024.1453145

**Published:** 2024-10-30

**Authors:** Shuo Yang, Min Huo, Zixuan Su, Fangfang Wang, Yongying Zhang, Cuihong Zhong, Yuxiang Shi

**Affiliations:** College of Life Science and Food Engineering, Hebei University of Engineering, Handan, China

**Keywords:** Quercetagetin, broilers, growth performance, antioxidant capacity, microbial community

## Abstract

This experiment aimed to investigate the effects of Quercetagetin (QG) on the growth performance, antioxidant capacity, and cecal microbiota of broilers. Two hundred and forty 21-day-old WOD168 broilers with similar body weights were randomly divided into five groups of six replicates each with eight chickens. The control group was fed a basal diet composed of corn and soybean meal, while the experimental groups received basal diets supplemented with 0, 10, 20, and 40 mg/kg QG, along with intraperitoneal injection of 20 mg/kg body weight Diquat (DQ). The experiment lasted for 21 days. The results showed that: (1) QG significantly alleviated the decrease in average daily feed intake and average daily gain induced by Diquat, reduced the elevation of serum ACTH content, and significantly increased GH content (*P* < 0.05); (2) QG supplementation significantly mitigated the decrease in serum CAT activity and duodenal GSH-Px activity induced by Diquat (*P* < 0.05), as well as the increase in MDA content (*P* < 0.05); additionally, QG significantly increased the gene expression levels of *GSH-Px, Nrf2*, and *Keap1* (*P* < 0.05); (3) Alpha and Beta diversity analysis revealed that QG supplementation significantly increased the cecal microbial OTUs and Chao1 index of broilers (*P* < 0.05). At the phylum level, compared with the Diquat group, the LQG group significantly decreased the relative abundance of Firmicutes (*P* < 0.05) and significantly increased the relative abundance of Bacteroidota (*P* < 0.05). At the genus level, compared with the CON group, the Diquat group significantly decreased the abundance of *Lactobacillus* and *Alistipes* (*P* < 0.05), while QG supplementation significantly alleviated the decrease in the abundance of *Lactobacillus* and *Alistipes* (*P* < 0.05). In conclusion, the addition of an appropriate amount (20 mg/kg) of QG to the diet can promote the growth of broilers, enhance antioxidant capacity, and improve intestinal health.

## 1 Introduction

In recent years, the intensification of poultry production systems has introduced a range of health challenges, with oxidative stress becoming a central issue. This condition arises from an imbalance between reactive oxygen species (ROS) production and the antioxidant defense mechanisms within the organism. The resulting oxidative stress impairs the health, growth performance, and overall productivity of poultry, particularly in high-density, intensive farming environments. Addressing the underlying mechanisms of oxidative stress and developing effective mitigation strategies are vital steps toward improving animal welfare and ensuring the long-term sustainability of poultry production systems.

The scale and intensification of breeding models has become the norm in modern animal husbandry. However, this approach has also led to the increasing emergence of various poultry health issues (Estévez, 2015). In intensive breeding environments, multiple factors such as pathological agents, unbalanced nutritional supply, and deficiencies in management can collectively trigger oxidative stress in poultry, leading to intestinal dysfunction and suboptimal gut health (Kong et al., 2022; Zheng et al., 2020). These issues not only affect the production performance of poultry but also result in a decline in the quality of livestock products, causing significant economic losses. To investigate oxidative stress in poultry, diquat (DQ), a bipyridyl herbicide, is commonly used to establish stress models due to its potent ability to generate ROS and induce oxidative damage across various tissues, including the liver and intestines (Chen et al., 2020). DQ toxicity is characterized by the production of highly reactive free radicals, such as superoxide anions (${\text{O}}_{2^{\cdot }}$-), through single-electron reduction and redox cycling. When the body's antioxidant defenses are overwhelmed, these excess free radicals lead to oxidative stress, resulting in mitochondrial dysfunction, disruption of intracellular homeostasis, inflammation, cell cycle arrest, and ultimately, cell death (Yu et al., 2022). Previous studies have demonstrated that intraperitoneal injection of 20 mg/kg body weight of DQ can successfully establish an oxidative stress model in broilers (Wu F. et al., 2023; Chen et al., 2020). This suggests that the selected dose effectively induces oxidative stress, providing a reliable experimental framework for further investigations into the physiological responses and underlying mechanisms of oxidative damage in poultry. Such a model is crucial for evaluating interventions aimed at mitigating oxidative stress, including nutritional strategies and antioxidant treatments, which may enhance poultry health and production efficiency under stress conditions. Numerous studies have confirmed that factors such as mycotoxins, pesticide compounds, high fat diets, nutrient deficiencies, and intestinal ischemia can all trigger severe oxidative stress in the poultry intestine, causing significant harm to animal health (Chen et al., 2022; Wang et al., 2022). The generation of oxidative stress can primarily be attributed to two key mechanisms: first, an imbalance between oxidative products and the antioxidant defense system within cells, disrupting the redox balance (Li et al., 2019); and second, the overproduction of ROS triggered by internal or external stressors. Under normal physiological conditions, animals have the ability to regulate the balance between oxidation and antioxidation, mitigating oxidative stress. However, when ROS scavenging mechanism fail to act promptly, excessive ROS can severely damage cell lipids, proteins, and DNA, disrupting cell signaling pathways and physiological functions, ultimately leading to a series of health problems (Chen et al., 2020). Thus, understanding and addressing oxidative stress-related intestinal health issues in poultry, particularly in intensive breeding environments, is therefore of significant importance for improving the productivity and product quality of livestock products.

The intestinal microbiota, as a dynamic and complex ecosystem, is influenced by numerous interactions among microbes, host, diet, and environment. These microbial communities play a crucial role in the development of host organ morphology, immune responses, metabolic processes, and overall health (Waite and Taylor, 2015). The functions of the gut microbiota extend beyond nutrient metabolism and absorption; it also plays a central role in maintaining intestinal barrier integrity, modulating immune system, and providing resistance against pathogen invasion (Bhagat et al., 2023). By regulating gut homeostasis, the microbiota helps prevent the occurrence of intestinal diseases in animals (Polansky et al., 2015). The balance of the gut flora is closely linked to the host's ability to resist pathogens, and plays a critical role in protecting the host from harm. Research has shown that incorporating plant-derived feed additives into poultry diets is an effective strategy for enhancing animal health and production performance (Li et al., 2022). Rich in a various of bioactive components, these additives not only improve animal growth performance but also to boost immune function, enhance antioxidant status, and stabilize the gut microbiota. Therefore, the inclusion of plant-derived feed additives in animal nutrition strategies that leverage their bioactive components to optimize gut microbiota, is an effective approach to improve animal health and production efficiency. This strategy also holds significant promise for promoting the sustainable development of the livestock industry.

Recently, natural plant extracts have garnered significant attention for maintaining the intestinal health of livestock and poultry due to their safety, stability, and lower potential for resistance development. Particularly, flavonoid compounds, as secondary metabolites of plants, have become a focus of research due to their abundant resources, diverse origins, and their potent anti-inflammatory, antibacterial, antioxidant, and gastrointestinal function-regulating properties (Wan et al., 2024). In livestock and poultry production, flavonoids positively impact intestinal health by promoting digestive and absorptive functions, enhancing intestinal structure integrity, regulating mucosal layers, boosting the expression of tight junction proteins, and reducing epithelial cell permeability. Quercetagetin (QG), chemically known as 3,3′,4′,5,6,7-hexahydroxyflavone (chemical formula C_15_H_10_O_8_), is one of the main active components extracted from marigold and belongs to the flavonol category of flavonoids (Wu F. Y. et al., 2023). Due to its structure containing six phenolic hydroxyl groups, it exhibits excellent antioxidant and antibacterial properties. Previous studies have shown that QG has a strong capability to scavenge DPPH·, ·OH, and O_2_.– radicals (Fuentes et al., 2021). Supplementing broiler diets with QG has been shown to significantly improve apparent digestibility, enhance the structural morphology of the duodenum and ileum, and enhance immune function. However, research on the effects of QG as a feed additive on the antioxidant function of the chicken intestine and gut microbiota remains limited (Wu et al., 2022). This study utilizes a DQ-induced oxidative stress model in broilers to investigate the effects of QG on growth performance, intestinal antioxidant capacity, and gut microbiota diversity. The objective is to elucidate its potential applications in livestock and poultry production, providing a scientific foundation and practical insights for improving production efficiency and promoting animal health.

## 2 Materials and methods

### 2.1 Experimental materials

QG was procured from the Chenguang Biotech Group Co., Ltd., with a purity exceeding 85%. Diquat (Diquat Dibromide Monohydrate, Aladdin, Lot#b2327176) was obtained from Shanghai Aladdin Biochemical Technology Co., Ltd. Glutathione peroxidase (GSH-Px), Catalase (CAT), Superoxide Dismutase (SOD), and Malondialdehyde (MDA) assay kits were purchased from Nanjing Jiancheng Bioengineering Institute. RNAiso Plus, SYBR Green qPCR Master Mix, and the SYBR Green PCR kit were obtained from Takara Biotechnology Co., Ltd. in Dalian.

### 2.2 Animal ethics statement

The experimental protocol was approved by the Animal Care and Use Committee of Hebei University of Engineering (Handan, China). The animal experiments were conducted in strict adherence to the guidelines outlined in the Care and Use of Animals (BER-YXY-2024006).

### 2.3 Experimental design, animal, and management

**Figure 1:**
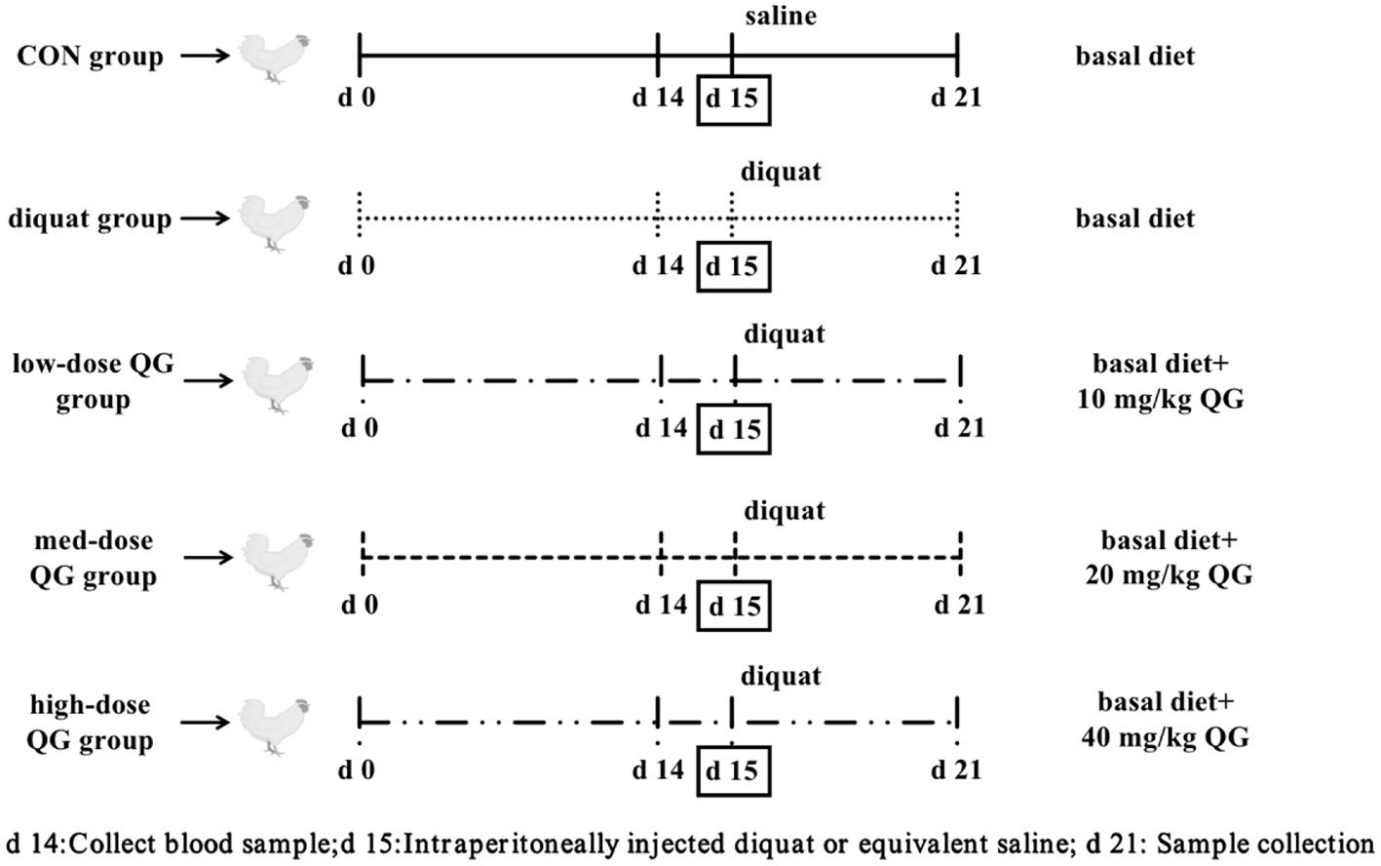
Broiler experiment flow diagram. DQ, Diquat; QG, Quercetagetin.

The experiment utilized a single-factor randomized design. A total of 240 21-day-old WOD168 broilers were selected and randomly assigned to five treatment groups, ensuring similar body weight across groups. The groups were as follows: the control group (CON), which was fed a basal diet and intraperitoneally injected with 0.9% saline; the Diquat group (DQ), which was fed a basal diet and intraperitoneally injected with Diquat; the low-dose QG group (LQG), which received 10 mg/kg QG added to the basal diet and was intraperitoneally injected with Diquat; the medium-dose QG group (MQG), which received 20 mg/kg QG added to the basal diet and was intraperitoneally injected with Diquat; and the high-dose QG group (HQG), which received 40 mg/kg QG added to the basal diet and was intraperitoneally injected with Diquat. Each treatment group had six replicates, with eight chickens per replicate. The experiment lasted for 21 days, with a pre-feeding period from days 1 to 14. On the 15th day, broilers in the Diquat treatment groups were intraperitoneally injected with a Diquat solution at 2 mL/kg body weight (Diquat solution concentration was 10 mg/mL in saline), while the control group received an equivalent volume of saline. The experimental design is referenced in Figure 1.

During the experimental period, all broilers were housed in fully automated, standardized poultry houses with *ad libitum* access to feed and water. The housing was structured as a three-tier cage system. The basal diet was a corn-soybean meal type, formulated into pelleted feed according to the nutritional recommendations of the NRC (1994) and the Chinese Nutrient Requirements of Poultry (NY/T 33-2004). The composition and nutritional levels of the basal diet are detailed in Table 1. All groups were managed under uniform husbandry and environmental conditions to ensure consistency throughout the experiment.

**Table 1:** Composition and nutrient level of the basal diet (as-feed basis), %.

**Items**	**Content**
**Ingredients**	
Corn	58.80
Soybean meal	33.80
Soybean oil	3.00
Dicalcium phosphate	1.80
Limestone	1.22
Salt	0.37
L-Lysine	0.03
DL-Methionine	0.07
Premix^a^	0.80
Choline	0.11
Total	100.0
**Nutrient levels** ^b^	
Metabolizable energy (MJ/kg)	12.62
Crude protein	19.65
Calcium	1.02
Available phosphorus	0.42
Lys	1.15
Met	0.40
Cys	0.36

^a^Premix provided the following per kilogram of diet: vitamin A: 9,000 IU, vitamin D3: 3,000 IU, vitamin E: 26 mg, vitamin K3: 1.20 mg, vitamin B: 13.00 mg, vitamin B2: 8.00 mg, vitamin B6: 4.40 mg, vitamin B12: 0.012 mg, nicotinic acid: 45 mg, folic acid: 0.75 mg, biotin: 0.20 mg, choline: 1,100 mg, calcium pantothenate: 15 mg, Fe: 100 mg, Cu: 10 mg, Zn: 108 mg, Mn: 120 mg, I: 1.5 mg, and Se: 0.35 mg.

^b^Crude protein is a measured value, while the others are all calculated values.

### 2.4 Sample collection and preparation

On day 21 of the trial, a broiler with an average weight representative of its group was selected from each replicate for blood collection. Blood samples were collected from the wing vein using vacuum blood collection tubes without anticoagulant. After allowing the samples to sit at room temperature for 30 min to facilitate clot formation, they were centrifuged at 3,000 × *g* for 15 min at 4°C. The resulting serum samples were then carefully collected and stored at −20°C for subsequent analysis of serum hormone levels and antioxidant indicators. Following blood collection, the chickens were euthanized by exsanguination from the neck, and the small intestine (duodenum, jejunum, and ileum) was harvested. A segment from the middle of each intestinal section was cleaned of its contents with saline, surface moisture was absorbed with filter paper, and the mucosa was scraped onto a slide, collected into 2 mL sterile tubes, and stored at −20°C for antioxidant indicator analysis. Another portion of the small intestinal tissue was rinsed with pre-chilled sterile saline solution to remove blood contamination, quickly frozen in liquid nitrogen, and then stored at −80°C for RNA extraction. Additionally, 2 g of cecal contents were collected into cryovials, flash-frozen in liquid nitrogen, and stored at −80°C for the analysis of microbial diversity.

### 2.5 Determination indexes and methods

#### 2.5.1 Growth performance

The broilers were weighed on days 14 and 21 of the trial. Feed intake was evaluated by measuring the total amount of feed supplied and the remaining feed at the end of each day, which facilitated the calculation of daily feed consumption. Key performance metrics, including Average Daily Gain (ADG), Average Daily Feed Intake (ADFI), and Feed-to-Gain ratio (F/G), were subsequently derived from these measurements for each experimental period. The health status of the broilers was observed daily, and the mortality rate was also recorded.

#### 2.5.2 Serum stress hormone indicators

The serum levels of ACTH, CORT and GH were determined using commercial ELISA kits from Genway Biotech, Inc., strictly following the procedures provided in the kit manuals.

#### 2.5.3 Serum and tissue sample antioxidant indexes

Following the method described by Shi et al. (2022), antioxidant indicators, including SOD, CAT, and GSH-Px, as well as the oxidative stress damage marker MDA, were measured in the samples using commercial assay kits (Nanjing Jiancheng Bioengineering Institute, Nanjing, China). The specific procedures were conducted strictly according to the instructions provided in the kit manuals.

#### 2.5.4 Expression level of antioxidant-related genes in small intestine

Total RNA from the small intestine was extracted using the Trizol method. RNA purity and concentration were assessed spectrophotometrically at 260 and 280 nm, and RNA integrity was evaluated by 1.5% agarose gel electrophoresis. Reverse transcription was performed using the TaKaRa Reverse Transcription Kit according to the manufacturer's instructions. Quantitative fluorescence PCR was conducted using Gen Star qPCR PreMix and the 7500 Real-Time PCR System. The total reaction volume was 20 μL, consisting of 10 μL 2 × RealStar Fast SYBR PCR Mix, 0.5 μL of each primer, 8 μL ddH_2_O, and 1 μL cDNA template. The reaction conditions were set as follows: pre-denaturation at 95°C for 30 s, denaturation at 95°C for 5 s, annealing at 60°C for 30 s, and extension at 72°C for 20 s, for a total of 45 cycles. In this experiment, β*-actin* served as the internal reference gene, and the relative expression levels of the *GSH-Px, CAT, SOD, Nrf2*, and *Keap1* genes were calculated using the 2^−Δ*ΔCt*^ method. Primers for the relevant genes were synthesized by Sangon Biotech Co., Ltd., with the primer sequences presented in Table 2.

**Table 2:** Genes and their primer sequences.

**Genes**	**Gene bank No**.	**Primer sequences, 5^′^-3^′^**	**Length (bp)**
*β-Actin*	NM_205518	F-GCCAACAGAGAGAAGATGACAC	118 bp
		R-GTAACACCATCACCAGAGTCCA	
*Nrf2*	NM_205117.1	F-GATGTCACCCTGCCCTTAG	215 bp
		R-CTGCCACCATGTTATTCC	
*SOD*	NM_205064.1	F-TTGTCTGATGGAGATCATGGCTTC	98 bp
		R-TGCTTGCCTTCAGGATTAAAGTGA	
*CAT*	NM_001031215.1	F-GTTGGCGGTAGGAGTCTGGTCT	182 bp
		R-GTGGTCAAGGCATCTGGCTTCTG	
*GSH-Px*	NM_001163245.1	F-CAAAGTTGCGGTCAGTGGA	136 bp
		R-AGAGTCCCAGGCCTTTACTACTTTC	
*Keap1*	XM_015274015.1	F-TGCCCCTGTGGTCAAAGTG	104 bp
		R-GGTTCGGTTACCGTCCTGC	

F, forward primer; R, reverse primer; β-Actin, beta-actin; Nrf2, nuclear factor erythroid-2-related factor 2; SOD, superoxide dismutase; CAT, catalase; GSH-Px, glutathione peroxidase; Keap1, kelch like ECH associated protein 1.

#### 2.5.5 Cecal microbiota

Following the method described by Li et al. (2021), cecal microbial DNA was extracted, the V3-V4 variable regions of the 16S rDNA were amplified, libraries were constructed, sequencing and species annotation were performed, and analyses were conducted to calculate the relative abundance of cecal microbial phyla and genera based on operational taxonomic units (OTUs), as well as Alpha and Beta diversity.

### 2.6 Statistical analysis of data

After being organized in Excel 2016, all data were subjected to one-way ANOVA using the SPSS 21.0 software for the analysis of variance. When significant differences in treatment effects were observed, Duncan's multiple range test was applied for *post-hoc* comparisons. Differences were considered significant at *P* < 0.05. The results of the experiment are presented as mean values with standard error of the mean (SEM).

## 3 Results

### 3.1 Growth performance

The effects of different concentrations of QG on the growth performance of broiler chickens are presented in Table 3. On the 14th day of the experiment, there were no significant differences among the groups in BW, ADFI, ADG, and F:G (*P* > 0.05). On the 21st day of the experiment, compared to the DQ group, both the LQG and MQG groups significantly increased the BW of broiler chickens (*P* < 0.05). Compared to the CON group, the DQ group significantly decreased ADFI (*P* < 0.01), while the QG treatment groups (LQG, MQG, and HQG) were significantly higher in ADFI than the DQ group (*P* < 0.01), and there were no significant differences in ADFI compared to the CON group (*P* > 0.05). Compared to the CON group, the DQ group significantly decreased ADG (*P* < 0.05), while the MQG and HQG groups were significantly higher than the DQ group (*P* < 0.05), with no significant differences compared to the CON group (*P* > 0.05). Furthermore, compared to the CON group, both the LQG and MQG groups significantly decreased F:G (*P* < 0.05).

**Table 3:** Effects of different concentrations of QG on growth performance of broilers.

	**Item**	**CON**	**DQ**	**LQG**	**MQG**	**HQG**	**SEM**	***P*-value**
14 d	BW (g)	1,108.85 ± 187.48	946.94 ± 21.46	1,221.50 ± 53.23	1,235.33 ± 58.23	1,198.00 ± 51.30	44.49	0.17
	ADFI (g)	87.19 ± 9.33	90.76 ± 18.35	89.83 ± 13.04	99.34 ± 8.93	93.72 ± 5.58	2.74	0.22
	ADG (g)	42.43 ± 8.59	36.53 ± 17.65	47.98 ± 2.29	49.51 ± 2.79	45.82 ± 2.53	2.41	0.47
	F:G	2.00 ± 0.08	2.02 ± 0.09	1.97 ± 0.08	1.95 ± 0.03	2.01 ± 0.11	0.02	0.70
21 d	BW (g)	1,537.67 ± 61.60^bc^	1,492.00 ± 69.66^c^	1,646.67 ± 40.61^ab^	1,674.67 ± 63.26^a^	1,510.33 ± 78.68^c^	24.25	0.02
	ADFI (g)	91.40 ± 12.69^A^	67.51 ± 27.02^B^	97.77 ± 5.62^A^	105.59 ± 5.37^A^	102.57 ± 4.86^A^	4.53	<0.01
	ADG (g)	57.19 ± 3.38^a^	48.86 ± 2.95^b^	53.57 ± 1.89^ab^	59.19 ± 3.26^a^	58.00 ± 5.39^a^	1.25	0.03
	F:G	1.90 ± 0.1^ab^	2.01 ± 0.28^a^	1.65 ± 0.06^bc^	1.57 ± 0.08^c^	1.86 ± 0.20^abc^	0.05	0.04

CON, control group; DQ, diquat group; LQG, low-dose quercetagetin group; MQG, medium-dose quercetagetin group; HQG, high-dose quercetagetin group; BW, body weight; ADG, average daily gain; ADFI, average daily feed intake; F:G, feed-to-gain ratio; SEM, standard error of the mean (*n* = 6).

In peer data, no letters or the same letters indicate no significant difference (*P* > 0.05), different lowercase letters indicate a significant difference (*P* < 0.05), and different uppercase letters indicate an extremely significant difference (*P* < 0.01).

### 3.2 Serum stress hormone indicators

The effects of different concentrations of QG on the serum stress hormone indicators of broiler chickens are presented in Table 4. On the 14th day of the experiment, there were no significant differences among the groups in ACTH, CORT, and GH (*P* > 0.05). On the 21st day of the experiment, compared to the CON group, the DQ group significantly increased the ACTH content (*P* < 0.05), while the ACTH content in the MQG group was significantly lower than that in the DQ group (*P* < 0.05), with no significant differences compared to the CON group (*P* > 0.05). Furthermore, compared to the CON group, the DQ group significantly decreased the GH content (*P* < 0.05), while both the LQG and MQG groups were significantly higher than the DQ group (*P* < 0.05), with no significant differences compared to the CON group (*P* > 0.05).

**Table 4:** Effects of different concentrations of QG on serum hormonal indicators in broilers.

	**Item**	**CON**	**DQ**	**LQG**	**MQG**	**HQG**	**SEM**	***P*-value**
14 d	ACTH (pg/mL)	17.09 ± 4.85	21.54 ± 9.17	17.07 ± 6.04	12.28 ± 3.70	15.89 ± 8.47	1.38	0.39
	CORT (ng/mL)	2.62 ± 0.41	3.71 ± 1.88	2.96 ± 1.35	2.51 ± 0.89	2.93 ± 0.67	0.21	0.44
	GH (ng/mL)	4.83 ± 0.52	4.43 ± 0.49	4.89 ± 0.37	5.06 ± 0.36	5.02 ± 0.53	0.35	0.16
21 d	ACTH (pg/mL)	17.95 ± 2.94^b^	27.38 ± 1.95^a^	18.70 ± 1.16^ab^	16.98 ± 1.71^b^	24.14 ± 1.51^ab^	1.66	0.04
	CORT (ng/mL)	3.09 ± 0.70	3.19 ± 0.24	2.82 ± 0.40	2.72 ± 0.35	3.02 ± 0.27	0.08	0.33
	GH (ng/mL)	5.12 ± 0.48^a^	4.49 ± 0.39^b^	5.26 ± 0.51^a^	5.54 ± 0.49^a^	4.87 ± 0.40^ab^	0.91	0.02

CON, control group; DQ, diquat group; LQG, low-dose quercetagetin group; MQG, medium-dose quercetagetin group; HQG, high-dose quercetagetin group; ACTH, adrenocorticotropic hormone; CORT, corticosterone; GH, growth hormone; SEM, standard error of the mean (*n* = 6).

In peer data, no letters or the same letters indicate no significant difference (*P* > 0.05), different lowercase letters indicate a significant difference (*P* < 0.05).

### 3.3 Serum and tissue sample antioxidant indexes

The effects of different concentrations of QG on the serum antioxidant indices of broiler chickens are presented in Table 5. On day 14 of the experiment, compared to the CON group, the MQG group showed a significant increase in SOD activity (*P* < 0.05); however, there were no significant differences in GSH-Px, CAT, and MDA activity or content among the groups (*P* > 0.05). On day 21 of the experiment, compared to the CON group, the DQ group significantly decreased CAT activity (*P* < 0.05), while the CAT activity in the LQG group was significantly higher than that in the DQ group (*P* < 0.05), with no significant differences compared to the CON group (*P* > 0.05). Moreover, compared to the CON group, the DQ group significantly increased MDA content (*P* < 0.05); however, the MDA content was significantly reduced in the LQG, MQG, and HQG groups compared to the DQ group (*P* < 0.05).

**Table 5:** Effects of different concentrations of QG on serum antioxidant indicators in broilers.

	**Item**	**CON**	**DQ**	**LQG**	**MQG**	**HQG**	**SEM**	***P*-value**
14 d	GSH-Px (U/mL)	3,895.7 ± 29.31	3,895.3 ± 23.45	3,820.4 ± 32.55	3,793.6 ± 31.79	3,217.8 ± 34.79	193.17	0.52
	CAT (U/mL)	4.92 ± 0.68	2.99 ± 0.15	4.41 ± 0.73	3.81 ± 0.48	3.07 ± 0.26	0.46	0.29
	SOD (U/mL)	338.07 ± 25.67^b^	343.80 ± 31.46^b^	369.66 ± 22.94^ab^	442.44 ± 30.51^a^	379.20 ± 41.22^ab^	19.37	0.03
	MDA (nmol/mL)	2.70 ± 0.14	2.91 ± 0.22	2.69 ± 0.39	2.10 ± 0.17	2.58 ± 0.30	0.22	0.46
21 d	GSH-Px (U/mL)	2,288.0 ± 247.9	2,215.0 ± 209.5	2,229.2 ± 198.6	2,186.3 ± 233.7	2,331 ± 221.5	269.09	0.88
	CAT (U/mL)	6.10 ± 0.52^a^	3.49 ± 0.48^b^	5.47 ± 0.57^a^	4.51 ± 0.59^ab^	4.77 ± 0.32^ab^	0.45	0.03
	SOD (U/mL)	358.49 ± 31.66	353.80 ± 29.53	332.10 ± 30.04	356.23 ± 31.77	346.49 ± 28.19	41.62	0.75
	MDA (nmol/mL)	2.54 ± 0.19^B^	3.28 ± 0.45^A^	1.85 ± 0.35^B^	1.96 ± 0.29^B^	3.06 ± 0.16^AB^	0.18	<0.01

CON, control group; DQ, diquat group; LQG, low-dose quercetagetin group; MQG, medium-dose quercetagetin group; HQG, high-dose quercetagetin group; GSH-Px, glutathione peroxidase; CAT, catalase; SOD, superoxide dismutase; MDA, malondialdehyde; SEM, standard error of the mean (*n* = 6).

In peer data, no letters or the same letters indicate no significant difference (*P* > 0.05), different lowercase letters indicate a significant difference (*P* < 0.05), and different uppercase letters indicate an extremely significant difference (*P* < 0.01).

The effect of different concentrations of QG on the intestinal antioxidant indices of broiler chickens is presented in Table 6. At day 21 of the experiment, compared to the CON group, the DQ group significantly decreased the duodenum GSH-Px activity (*P* < 0.05). In contrast, compared to the DQ group, the LQG, MQG, and HQG groups significantly increased the GSH-Px activity (*P* < 0.05). Additionally, compared to the CON group, the DQ group significantly increased the MDA content (*P* < 0.05). However, the MDA content in the LQG group was significantly lower than that in the DQ group (*P* < 0.05), and there was no significant difference compared to the CON group (*P* > 0.05). Furthermore, compared to the DQ group, the MQG and HQG groups significantly increased the jejunum GSH-Px activity (*P* < 0.05). Similarly, compared to the CON group, the DQ group significantly increased the MDA content (*P* < 0.05), while the MDA content in the MQG and HQG groups was significantly lower than that in the DQ group (*P* < 0.05). Finally, compared to the CON group, the DQ group significantly increased the ileum MDA content (*P* < 0.05), while the MDA content in the LQG and MQG groups was significantly lower than that in the DQ group (*P* < 0.05).

**Table 6:** Effects of different concentrations of QG on small intestine antioxidant indicators in broilers.

	**Item**	**CON**	**DQ**	**LQG**	**MQG**	**HQG**	**SEM**	***P*-value**
Duodenum	GSH-Px (U/mg prot.)	6.69 ± 0.53^ab^	4.97 ± 0.49^c^	7.65 ± 0.91^ab^	9.05 ± 1.43^a^	8.93 ± 0.69^a^	0.76	0.02
	CAT (U/mg prot.)	1.22 ± 0.11	1.33 ± 0.25	1.11 ± 0.20	1.49 ± 0.15	1.37 ± 0.26	0.08	0.11
	SOD (U/mg prot.)	358.7 ± 30.17	308.2 ± 28.95	348.4 ± 40.97	373.3 ± 42.11	319.6 ± 45.85	27.1	0.32
	MDA (nmol/mg prot.)	0.74 ± 0.06^b^	1.18 ± 0.25^a^	0.66 ± 0.02^b^	0.84 ± 0.03^ab^	0.81 ± 0.05^ab^	0.11	0.03
Jejunum	GSH-Px (U/mg prot.)	6.69 ± 0.53^ab^	4.97 ± 0.79^b^	5.65 ± 0.66^b^	9.05 ± 0.81^a^	8.15 ± 0.88^a^	0.76	0.04
	CAT (U/mg prot.)	0.95 ± 0.07	1.20 ± 0.09	0.90 ± 0.05	1.10 ± 0.16	1.09 ± 0.11	0.16	0.11
	SOD (U/mg prot.)	255.43 ± 20.17	259.73 ± 28.67	292.43 ± 32.98	303.03 ± 28.15	285.16 ± 33.69	27.79	0.32
	MDA (nmol/mg prot.)	0.17 ± 0.01^b^	0.72 ± 0.08^a^	0.42 ± 0.02^ab^	0.29 ± 0.01^b^	0.39 ± 0.01^b^	0.14	0.02
Ileum	GSH-Px (U/mg prot.)	19.51 ± 2.03	19.79 ± 2.36	20.11 ± 3.58	16.28 ± 1.99	19.28 ± 1.71	3.50	0.53
	CAT (U/mg prot.)	2.07 ± 0.17	2.50 ± 0.33	2.74 ± 0.27	2.32 ± 0.19	1.98 ± 0.27	0.36	0.27
	SOD (U/mg prot.)	442.5 ± 40.18	465.1 ± 55.06	491.9 ± 51.13	472.8 ± 39.88	452.8 ± 44.05	38.62	0.49
	MDA (nmol/mg prot.)	0.60 ± 0.02^b^	0.93 ± 0.06^a^	0.70 ± 0.09^b^	0.63 ± 0.03^b^	0.87 ± 0.09^ab^	0.06	0.02

CON, control group; DQ, diquat group; LQG, low-dose quercetagetin group; MQG, medium-dose quercetagetin group; HQG, high-dose quercetagetin group; GSH-Px, glutathione peroxidase; CAT, catalase; SOD, superoxide dismutase; MDA, malondialdehyde; SEM, standard error of the mean (*n* = 6).

In peer data, no letters or the same letters indicate no significant difference (*P* > 0.05), different lowercase letters indicate a significant difference (*P* < 0.05).

### 3.4 Expression level of the antioxidant-related genes in the intestines

**Figure 2:**
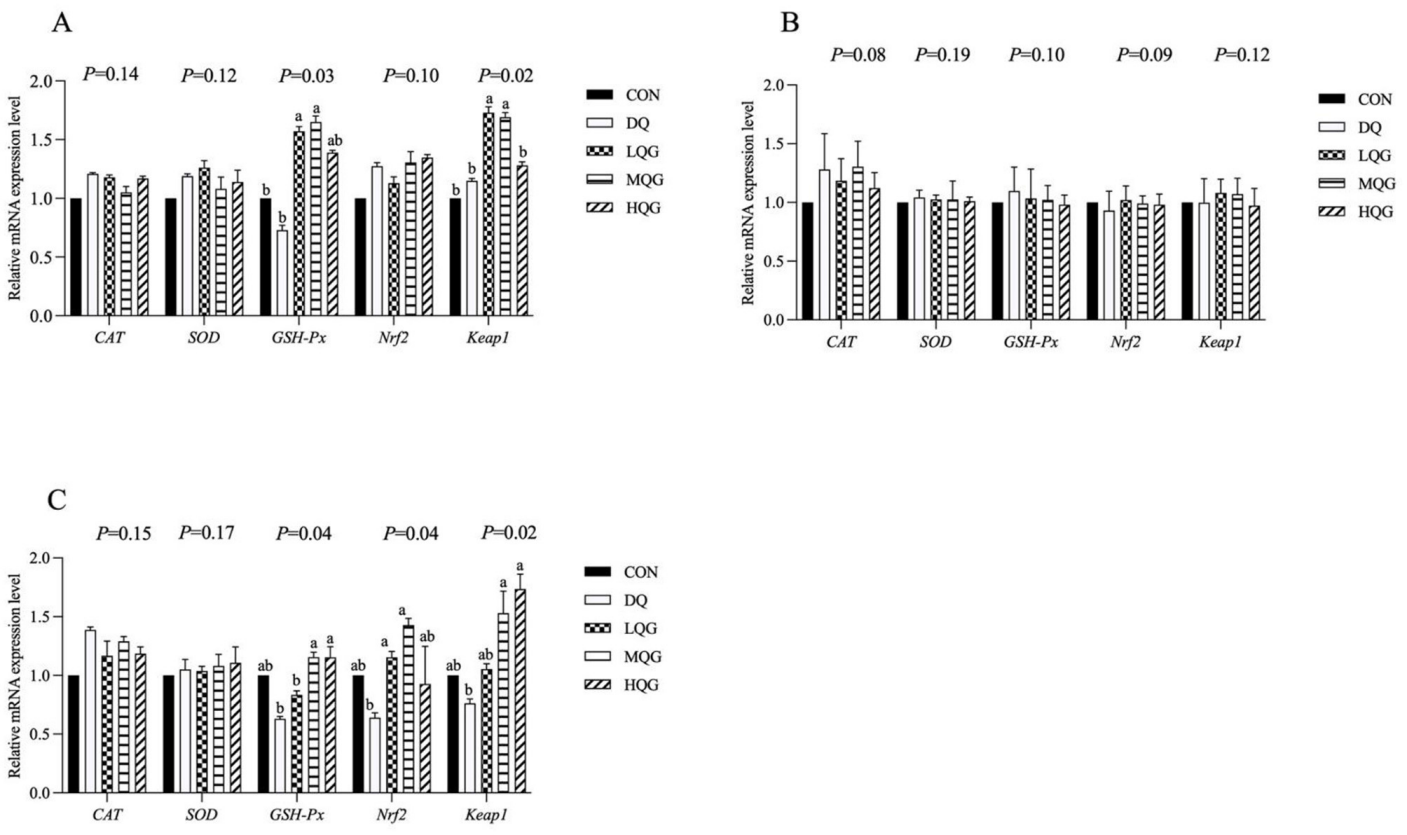
Effects of diets with different concentrations QG on antioxidant-related gene expression in the small intestine of broilers challenged with DQ (21 d). CON, control group; DQ, diquat group; LQG, low-dose quercetagetin group; MQG, medium-dose quercetagetin group; HQG, high-dose quercetagetin group. **(A)** Duodenum; **(B)** jejunum; **(C)** ileum. Bars represent the means ± standard deviation (SD) (*n* = 6). ^a, b^Different letters in the shoulder markers in the table indicate significant differences between groups (*P* < 0.05).

The effects of different concentrations of QG on the expression of antioxidant-related genes in the intestines of broilers are shown in Figure 2. At day 21 of the experiment, compared to the DQ group, both the low and medium QG (LQG and MQG) groups exhibited a significant increase in the expression of *GSH-Px* and *Keap1* mRNAs in the duodenum (*P* < 0.05). In the ileum, compared to the DQ group, the LQG and MQG groups showed a significant increase in the expression of *GSH-Px* and *Nrf2* mRNA (*P* < 0.05), and both the MQG and HQG groups exhibited a significant increase in the expression of *Keap1* mRNA (*P* < 0.05). However, in the jejunum, there were no significant differences in the mRNA expression of *CAT, SOD, GSH-Px, Nrf2*, and *Keap1* among the groups (*P* > 0.05).

### 3.5 Cecal microbial community diversity

**Figure 3:**
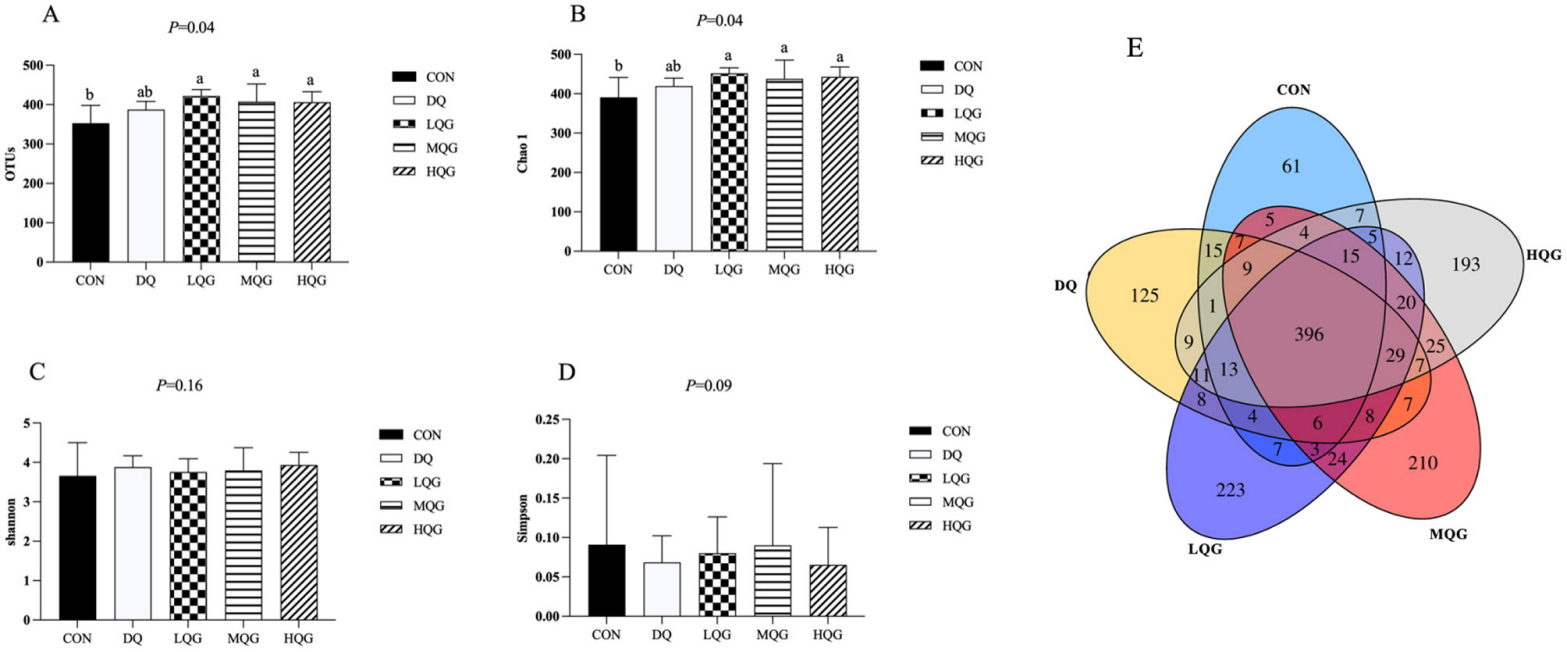
Effects of diets with different concentrations of QG on cecal microbial alpha diversity of broilers challenged with DQ. CON, control group; DQ, diquat group; LQG, low-dose quercetagetin group; MQG, medium-dose quercetagetin group; HQG, high-dose quercetagetin group. **(A)** OTUs; **(B)** Chao1 index; **(C)** Shannon index; **(D)** Simpson index; **(E)** Venn diagram of OTU. Bars represent the means ± standard deviation (SD) (*n* = 6). ^a, b^Different letters in the shoulder markers in the table indicate significant differences between groups (*P* < 0.05).

The effects of different concentrations of QG on the species richness and diversity of the cecal microbiota in broilers are shown in Figure 3. As indicated in Figures 3A, B, compared to the CON group, the operational taxonomic units (OTUs) and Chao1 indices were significantly increased in the QG treatment groups (LQG, MQG, and HQG) (*P* < 0.05). However, there were no significant differences in the Shannon and Simpson indices among the groups (*P* > 0.05) (Figures 3C, D). In Figure 3E, the Venn diagram displays the distribution of OTUs in the cecal contents of the experimental groups and the CON group. A total of 396 OTUs were shared across all groups. The CON group contained 61 unique OTUs, while the DQ, LQG, MQC, and HQG groups had 125, 223, 210, and 193 unique OTUs, respectively.

**Figure 4:**
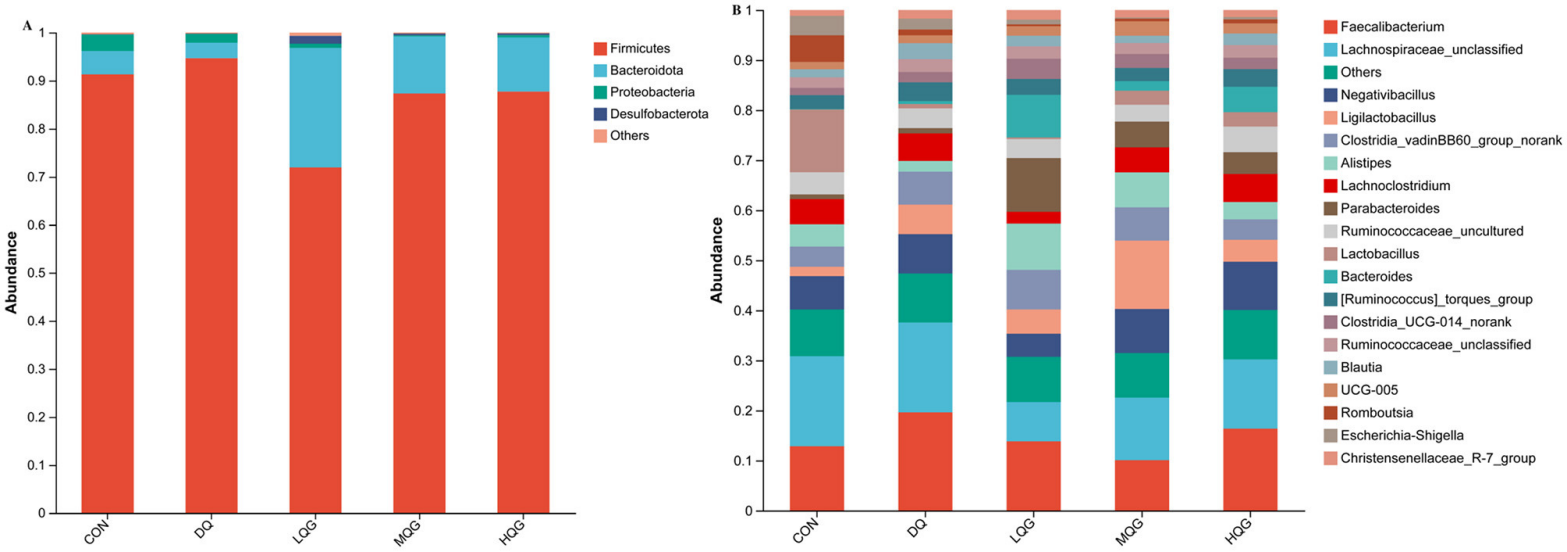
Effects of different concentrations of QG in the diet on the relative abundance of cecal microbiota at the phylum **(A)** and genus **(B)** levels in broilers challenged with DQ (*n* = 6). CON, control group; DQ, diquat group; LQG, low-dose quercetagetin group; MQG, medium-dose quercetagetin group; HQG, high-dose quercetagetin group. Cecal microbiota composition at the phylum **(A)** and genus **(B)** levels of broilers.

The 16S rRNA sequencing results revealed that, at the phylum level, the main bacterial were Firmicutes, Bacteroidota, Proteobacteria, and Desulfobacterota (Figure 4A). Among them, Firmicutes and Bacteroidota were the predominant phyla, collectively accounting for over 80% of the total microbial community. Compared to the DQ group, the LQG group significantly altered the relative abundance of Firmicutes and Bacteroidota, with a decrease in Firmicutes (*P* < 0.05) and an increase in Bacteroidota (*P* < 0.05). At the genus level (Figure 4B), compared to the CON group, the DQ group exhibited a significant increase in the relative abundance of *Faecalibacterium* (*P* < 0.05). Compared to the DQ group, the LQG group showed a significant reduction in the relative abundance of *Faecalibacterium* (*P* < 0.05). Additionally, both the LQG and MQG groups significantly reduced the relative abundance of *Lachnospiraceae_unclassified* (*P* < 0.05). Notably, the relative abundance of *Ligilactobacillus* in the MQG group was significantly higher than in the DQ group (*P* < 0.05) (Table 7).

**Table 7:** Effects of different concentrations of QG in the diet on the relative abundance of cecal microbiota at the phylum and genus levels in broilers challenged with DQ.

	**Item**	**CON**	**DQ**	**LQG**	**MQG**	**HQG**	**SEM**	***P*-value**
Phylum	Firmicutes	0.913 ± 0.04^A^	0.947 ± 0.02^A^	0.719 ± 0.02^B^	0.873 ± 0.02^A^	0.877 ± 0.02^A^	0.028	<0.01
	Bacteroidota	0.049 ± 0.00^B^	0.032 ± 0.00^B^	0.249 ± 0.02^A^	0.119 ± 0.01^B^	0.113 ± 0.01^B^	0.014	<0.01
Genus	*Faecalibacterium*	0.113 ± 0.00^ab^	0.190 ± 0.01^a^	0.121 ± 0.01^ab^	0.048 ± 0.00^b^	0.143 ± 0.02^ab^	0.089	0.03
	*Lachnospiraceae_unclassified*	0.159 ± 0.00^a^	0.152 ± 0.01^a^	0.069 ± 0.00^b^	0.106 ± 0.01^ab^	0.121 ± 0.02^ab^	0.049	0.02
	*Negativibacillus*	0.059 ± 0.00^ab^	0.067 ± 0.00^ab^	0.040 ± 0.00^b^	0.074 ± 0.01^ab^	0.084 ± 0.01^a^	0.031	0.02
	*Lactobacillus*	0.111 ± 0.01^a^	0.007 ± 0.01^c^	0.003 ± 0.00^c^	0.024 ± 0.00^b^	0.025 ± 0.00^b^	0.001	0.03

CON, control group; DQ, diquat group; LQG, low-dose quercetagetin group; MQG, medium-dose quercetagetin group; HQG, high-dose quercetagetin group; SEM, standard error of the mean (*n* = 6).

In peer data, no letters or the same letters indicate no significant difference (*P* > 0.05), different lowercase letters indicate a significant difference (*P* < 0.05), and different uppercase letters indicate an extremely significant difference (*P* < 0.01).

**Figure 5:**
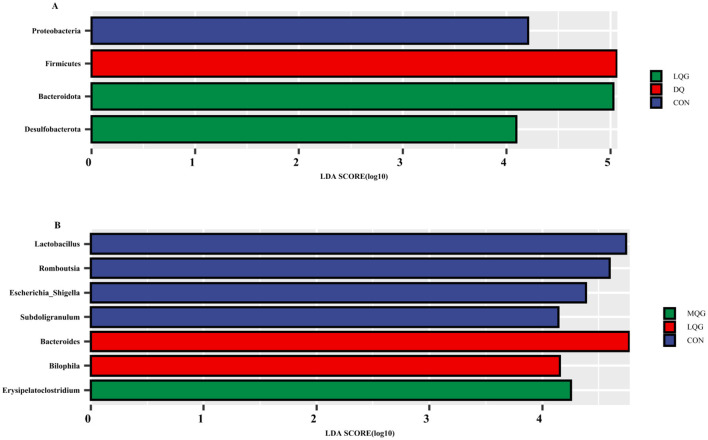
Linear discriminant analysis (LDA) effect size (LEfSe) analysis of the cecal microbiota (*n* = 6). CON, control group; DQ, diquat group; LQG, low-dose quercetagetin group; MQG, medium-dose quercetagetin group; HQG, high-dose quercetagetin group. Phylum-level LDA bar chart **(A)** and genus-level LDA bar chart **(B)** display the LDA scores generated for differentially abundant microbiota (LDA > 4, *P* < 0.05).

**Figure 6:**
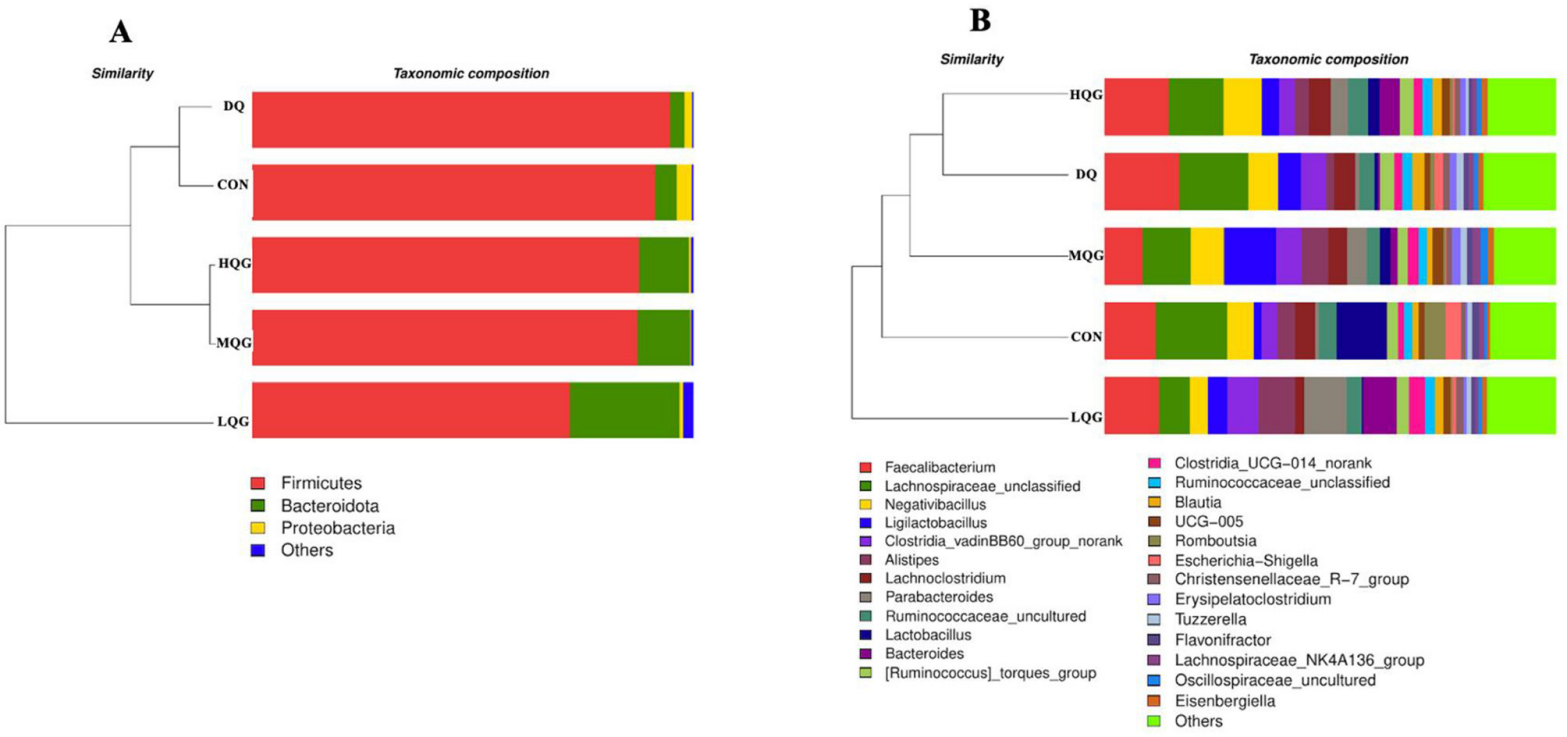
Effects of diets with different concentrations of QG on the microbial community barplot with cluster tree of broilers challenged with DQ (n = 6). CON, control group; DQ, diquat group; LQG, low-dose quercetagetin group; MQG, medium-dose quercetagetin group; HQG, high-dose quercetagetin group. Barplot of microbial communities with hierarchical clustering trees at the phylum level **(A)** and genus level **(B)**.

The linear discriminant analysis (LDA = 4) effect size (LEfSe) algorithm was employed to analyze the taxonomic abundance of the microbiota. The results at the phylum and genus levels are shown in Figure 5. At the phylum level (Figure 5A), the abundance of Proteobacteria was elevated in the CON group, while Firmicutes significantly increased in the DQ group, and Bacteroidota and Desulfobacterota were notably enriched in the LQG group. At the genus level (Figure 5B), the CON group exhibited significant enrichment of *Lactobacillus, Romboutsia, Escherichia-Shigella*, and *Subdoligranulum*, while the LQG group showed a marked increase in *Bacteroides* and *Bilophila*, and *Erysipelatoclostridium* was significantly elevated in the MQG group. The combined analysis of the sample clustering tree and bar chart (Figure 6) revealed that, at the phylum level (Figure 6A), the microbial composition of the DQ and CON groups exhibited similarities, while the HQG and MQG groups also showed a similar composition. At the genus level (Figure 6B), the HQG and DQ groups demonstrated similarity, whereas the MQG, CON, and LQG groups displayed distinct differences in microbial composition.

### 3.6 Correlation analysis between the growth performance, oxidative function indicators, and cecal microbiota

**Figure 7:**
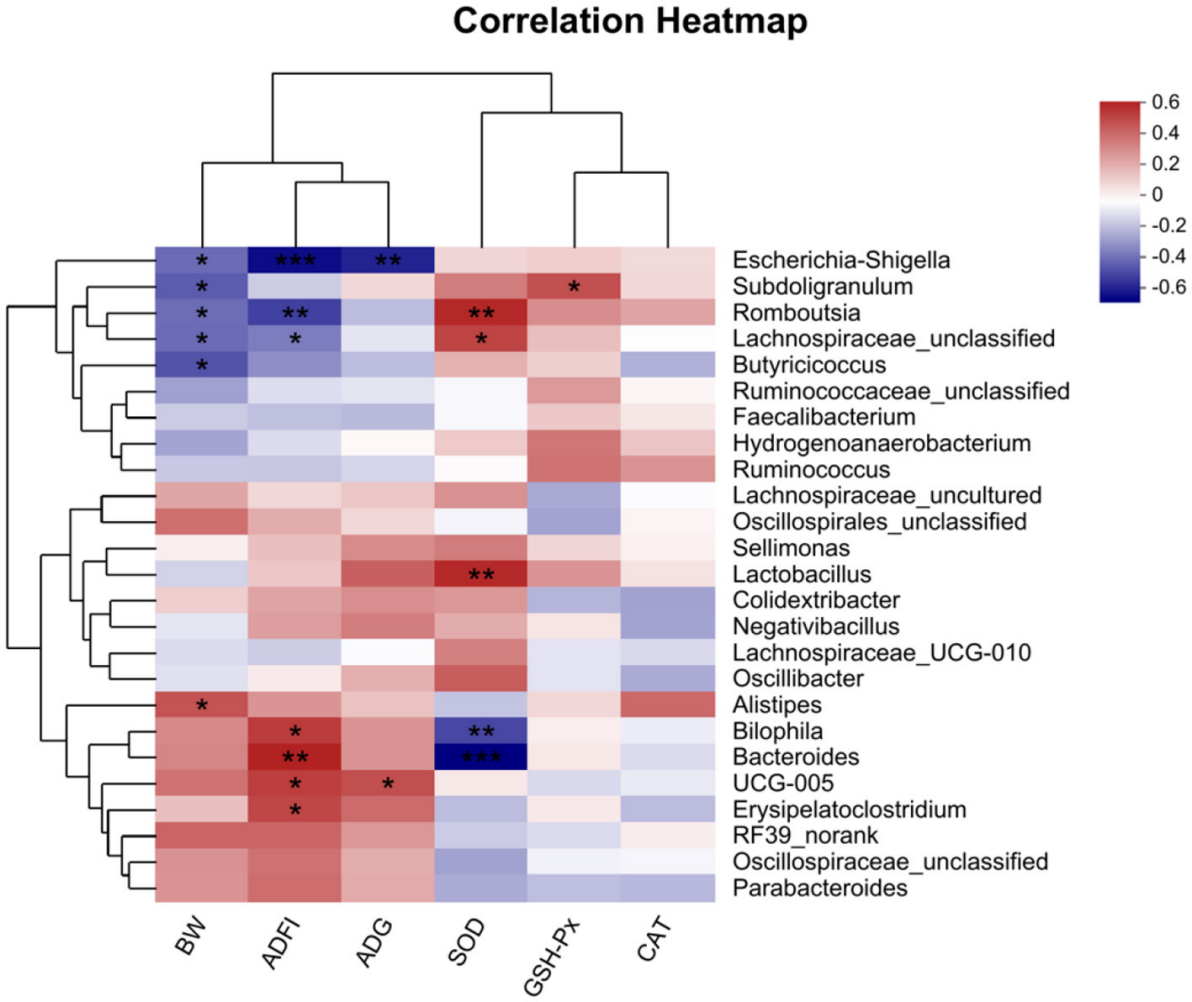
Correlation analyses between the Growth performance, oxidative function indicators and top 50 cecal bacterial genera (*n* = 6). The red squares represent the positive correlations while blue ones represent the negative correlations. *P < 0.05; **P < 0.01; ***P < 0.001. BW, body weight; ADFI, average daily feed intake; ADG, average daily gain; GSH-Px, glutathione peroxidase activity; CAT, catalase activity; SOD, superoxide dismutase activity.

The correlation between intestinal microbial proportions at the genus level and growth performance, as well as serum oxidative status among five groups, is illustrated in Figure 7. The results indicate a significant negative correlation between BW and *Escherichia-Shigella, Subdoligranulum, Romboutsia, Butyricicoccus*, and *Lachnospiraceae_unclassified*, while a significant positive correlation was observed with *Alistipes*. ADFI was significantly negatively correlated with *Escherichia-Shigella, Romboutsia*, and *Lachnospiraceae_unclassified*, and positively correlated with *Erysipelatoclostridium, UCG-005, Bilophila*, and *Bacteroides*. ADG exhibited a significant positive correlation with *UCG-005*. Additionally, GSH-Px showed a significant positive correlation with *Subdoligranulum*. SOD demonstrated significant positive correlations with *Romboutsia, Lactobacillus*, and *Lachnospiraceae_unclassified*, while showing significant negative correlations with *Bacteroides*, and *Bilophila*.

## 4 Discussion

Oxidative stress has extremely detrimental effects on the growth performance of poultry, while excessive antibiotics use not only poses significant threats to public health but also limits the sustainable development of animal husbandry (Lee et al., 2019). In this context, the search for green and safe alternatives to antibiotics has become a key focus in animal nutrition research. QG, a major component of extracts from *Tagetes erecta L*., belongs to the flavanol class and exhibits a range of biological activities, including antioxidant, anti-inflammatory, antibacterial properties, and the regulation of lipid metabolism (Wang et al., 2016). Although its application in poultry farming has been less frequently reported, flavonoid compounds have been shown to effectively enhance the growth performance of livestock and poultry. This study showed that intraperitoneal injection of diquat significantly reduced the BW of the broilers. This observation is consistent with previous studies indicating that diquat induces oxidative stress in broilers, which leads to a pronounced reduction in both ADG and ADFI (Chen et al., 2021). The phenomenon of diquat-induced oxidative stress seems to cause a redistribution of nutrients within the body, reallocating energy and glucose to physiological processes aimed at counteracting stress. This shift in nutrient allocation reduces feed intake, suppresses growth, and ultimately diminishes production efficiency. The mechanism underlying these effects is likely related to oxidative damage at the cellular level, particularly mitochondrial dysfunction, which not only impairs metabolic efficiency but also disrupts nutrient absorption. However, this experiment found that the addition of 20 and 40 mg/kg of QG significantly alleviated the BW decrease caused by diquat, with comparable effects to the basal diet group, and it had no adverse effects on the average daily feed intake or the feed conversion ratio of the broilers. This result suggests that adding quercetin glycoside to feed can improve the production performance of broiler chickens, which may be closely related to the antioxidant capacity and other biological activities of quercetin glycoside.

The levels of CORT and ACTH in serum are common indicators of stress, reflecting the physiological stress status of poultry (Guerreiro et al., 2016). When poultry are under stress, cytokines enter the central nervous system, activating the hypothalamic-pituitary-adrenal (HPA) axis, which leads to a rapid increase in ACTH and glucocorticoids (GC) in the blood. The release of ACTH stimulates the adrenal cortex to synthesize and release steroids, promoting the conversion of cholesterol into CORT, thus raising serum CORT levels (Cheng et al., 2019). Elevated CORT levels enhance the breakdown of nutrients to meet the body's physiological demands, resulting in alterations in nutrient distribution (Liu et al., 2015). Our study found that diquat stimulation significantly increased the concentration of the adrenocorticotropic hormone ACTH in the serum of broilers. This finding is consistent with the results of Li et al. (2015), who demonstrated that LPS stimulation similarly elevated serum ACTH levels while markedly reducing growth hormone (GH) levels in broilers. The observed increase in ACTH combined with the decrease in GH levels indicates that DQ-induced oxidative stress adversely impacts the growth performance of broilers. This impairment is most likely due to disruption of metabolic processes and nutrient absorption, as well as increased energy expenditure required to counteract the physiological stress induced by DQ. The elevation of ACTH suggests activation of theHPA axis, leading to enhanced glucocorticoid release, which in turn affects growth and metabolic efficiency. The concurrent reduction in GH further underscores the detrimental effects of oxidative stress on broiler growth, robust evidence for the role of DQ in inducing a stress response. Moreover, addressing the negative effects of oxidative stress and stress-induced hormonal imbalances, such as those observed with DQ stimulation, is crucial for improving broiler growth performance. Recent studies have highlighted the potential of medicinal plants in mitigating such adverse effects, primarily through their immune-enhancing properties. These plants, rich in secondary metabolites like flavonoids, polysaccharides, and polyphenols, have been shown to modulate the immune system and reduce oxidative stress (Song et al., 2018). By bolstering the immune response and counteracting oxidative damage, these natural compounds may help restore hormonal balance and metabolic efficiency in broilers. Consequently, incorporating medicinal plant extracts into broiler diets may offer a viable strategy to improve growth performance and overall productivity, aligning with efforts to enhance poultry health and mitigate the detrimental impacts of oxidative stress.

The redox state largely reflects the health status of an organism, and livestock and poultry typically possess various antioxidant enzymes to regulate the balance of redox reactions (Pisoschi and Pop, 2015; Doan et al., 2020). Research has demonstrated that flavonoid compounds can enhance the activity of antioxidant enzymes by activating the antioxidant system, thereby alleviating oxidative stress and enhancing the body's antioxidant capacity (Yuan et al., 2017). QG has been extensively studied in mice for its significant antioxidant properties and its positive effects on intestinal barrier function, yet studies in broilers are relatively scarce. Wu F. Y. et al. (2023) observed that dietary QG significantly alleviated the reduction in GSH-Px activity and the increase in MDA content in rabbit serum induced by zearalenone. Our study focused on the effects of dietary QG on the antioxidant performance of broilers, evaluating its impact through measurement of oxidative products in serum and intestines, key antioxidant enzymes, and the expression of genes involved in the antioxidant response. Our experimental results showed that adding 20 mg/kg of QG to the basic diet significantly increased SOD activity in serum. Additionally, feeding QG significantly mitigated the reduction in CAT activity and the increase in MDA content caused by DQ in broiler serum. These results are consistent with the findings of Wu et al. (2022), where QG supplementation also significantly increased GSH-Px activity and reduced MDA content in broiler serum. These observations suggest that adding QG to the feed enhance the activity of antioxidant enzymes and reduces lipid peroxidation products in broiler serum, thereby improving the body's antioxidant capacity and fostering a healthier environment for the growth and development of poultry.

Recent studies have indicated that redox processes begin in the intestine (Salvador et al., 2016). Nutrients absorbed into the small intestine contain numerous antioxidants but also various potential pro-oxidants, the intake of which may cause oxidative damage to the intestinal tract. Thus, maintaining redox balance in the intestines is crucial for overall health, as the intestinal mucosa is particularly vulnerable to oxidative damage induced by oxidants. For instance, in poultry and other animals, oxidized fats or lipids in aged grains can trigger severe oxidative stress in the intestines (Tang et al., 2019). Additionally, extensive research has shown that oxidative stress is frequently associated with heat stress or diquat stimulation. Zha et al. (2023) reported that diquat reduced T-AOC, CAT, SOD, and GSH-Px activities in a linear or quadratic manner, while increasing MDAaccumulation in serum, liver, and/or jejunum. Our experimental results demonstrated that the stimulation by diquat significantly reduced GSH-Px activity in the duodenum and significant increased MDA content in the small intestine (duodenum, jejunum, and ileum). Therefore, the judicious selection of dietary additives can help maintain ROS homeostasis and sustain intestinal health, thereby promoting healthy growth in livestock and poultry. Previous studies have demonstrated that plant extracts (Liu et al., 2018), probiotic (Song et al., 2014), and betaine (Saeed et al., 2017) can significantly enhance the antioxidant function of the intestinal tract in broilers. Research by Bakar et al. (2019) found that feeding naringin or hesperidin extracts significantly reduced MDA content in rat small intestine and notably increased SOD and GSH-Px activity in the intestine, alleviating symptoms of colitis. Moreover, our previous experiments found that *Artemisia* flavonoids possess excellent radical-scavenging abilities and antioxidant effects, which can enhance the activity of antioxidant enzymes (CAT, SOD, and GSH-Px) in the small intestine of broiler chickens, thereby improving the intestinal tissue's antioxidant capacity (Zhang P. F. et al., 2020; Yang et al., 2021). This study found that feeding QG significantly mitigated the reduction in GSH-Px activity and the increase in MDA content induced by diquat in broiler chicken small intestine. This indicates that feeding QG can protect the small intestine from lipid peroxidation-induced damage and help maintain redox homeostasis, thus enhancing the antioxidant function of the broiler's intestine. Integrating these results, we hypothesize that QG in the diet can enhance the antioxidant capacity of broiler chickens by increasing enzymatic activity and directly scavenging hydroxyl and superoxide anions in the small intestine, thereby maintaining intestinal redox stability, but the specific mechanisms involved require further investigation.

Antioxidant systems are fundamental to cellular defense against oxidative stress, relying on both enzymatic and non-enzymatic components. The enzymatic antioxidant system includes key enzymes such as SOD, CAT, and GSH-Px, forming the first line of defense against oxidative stress. The expression and activity of these antioxidant enzymes are primarily regulated by nuclear factor erythroid 2-related factor 2 (Nrf2). Under conditions without oxidative stress, Nrf2 forms an inactive complex with Keap1 protein in the cytoplasm. However, when cells encounter oxidative stress, Nrf2 dissociates from Keap1 and translocates to the nucleus, activating the expression of antioxidant response genes (Xing et al., 2021). Studies have shown that certain flavonoid compounds, such as QG, can enhance the activity of antioxidant enzymes by activating the Nrf2/Keap1 signaling pathway, effectively mitigating oxidative stress. This research further explores the effects of QG on the expression of antioxidant genes in the small intestine of broiler chickens and its potential molecular mechanisms (Sun et al., 2020). The experimental results indicate that the addition of QG to the feed significantly increased the mRNA levels of *GSH-Px* and *Keap1* in the duodenum, as well as *GSH-Px* and *Nrf2* mRNA levels in the ileum of broiler chickens. These findings suggest that QG may enhance the intrinsic antioxidant defense mechanisms of intestinal cells, improving their adaptive response to oxidative stress. Moreover, these results underscore the central role of the Nrf2/Keap1 signaling pathway in regulating the antioxidant defense response of the intestines. By activating this pathway, QG not only enhances the expression of antioxidant enzymes but may also promote the synthesis of other antioxidant-related proteins, providing comprehensive cellular protection. According to research by Sahin et al. (2023), QG enhances the activity of antioxidant enzymes in tissues by upregulating the Keap1/Nrf2/antioxidant response element (ARE) pathway, thereby alleviating oxidative damage in the retinas of mice. Additionally, other studies have found that QG activates the Nrf2/HO-1 signaling pathway in the liver of broiler chickens, increasing the activity of downstream key factors (HO-1) and antioxidant enzymes (SOD), and reducing oxidative damage caused by the herbicide paraquat (Chen et al., 2020). However, the current study found no significant differences in the mRNA expression of *CAT, SOD, GSH-Px, Nrf2*, and *Keap1* in the jejunum across all groups. This finding suggests that while certain parts of the intestine enhance antioxidant defense by upregulating related gene expression in response to specific antioxidants, other parts may respond differently or require different dosages and treatment durations to exhibit effects.

In addition to its well-documented effects on the Nrf2/Keap1 pathway, QG may influence other critical antioxidant defense mechanisms, including the NF-κB and MAPK signaling pathways. These pathways play pivotal roles in regulating cellular responses to oxidative stress and are intricately interconnected with Nrf2. Importantly, they can also exhibit negative feedback mechanisms that modulate Nrf2 activity. The interplay between NF-κB, MAPK, and Nrf2 may provide an additional layer of protection by refining and enhancing cellular adaptive responses to oxidative challenges (Zhou et al., 2018; Wardyn et al., 2015). This synergistic interaction could lead to a more robust cellular defense strategy. For example, activation of NF-κB can upregulate pro-inflammatory cytokines, which may, in turn, influence the expression of Nrf2 target genes. Similarly, the MAPK pathway can modulate Nrf2 activity through phosphorylation, enhancing its transcriptional efficacy under oxidative stress conditions (Hanson and Batchelor, 2022). Furthermore, QG impact may extend beyond enzymatic antioxidants to non-enzymatic systems, notably glutathione (GSH), one of the most abundant intracellular antioxidants. By promoting GSH synthesis or recycling via modulation of related enzymes, QG may enhance the cell's ability to counteract ROS. This is supported by our observation of increased *GSH-Px* mRNA levels, suggesting that QG reinforces the GSH system, a vital component of the antioxidant defense network. These findings underscore the multifaceted role of QG in maintaining cellular redox homeostasis and protecting against oxidative damage across multiple levels. Future research needs to further explore the impact of QG on other antioxidant pathways and how these pathways work synergistically to enhance the overall antioxidant capacity and health status of broiler chickens. This is vital for developing more effective feed additives to improve the production efficiency and animal welfare in the poultry industry.

The cecum is an important site in the body for water regulation, urea recycling, and carbohydrate fermentation, and is crucial for the intestinal nutritional health of broiler chickens. Studies have shown that the diversity and richness of intestinal microbiota are closely related to the likelihood of broiler chicken disease occurrence (Guo et al., 2010). In microbial alpha-diversity analysis, the Chao1 index reflects the abundance of the microbial communities, with a higher Chao1 index indicating greater community abundance. The Shannon and Simpson indices reflect community diversity. Specifically, a higher Shannon index and a lower Simpson index indicate greater species richness. Typically, higher microbial diversity indices indicate a more stable microbial community that is more resistant to disruptions. Our study found that the OTUs and Chao1 indices of the QG treatment groups (LQG, MQG, and HQG) were significantly higher than those of the CON group. It is speculated that the addition of QG to the diet may alter microbial evenness, increase microbial abundance, and enhance diversity. The cecum is the site with the highest microbial content in the poultry intestine. Studies have shown that Firmicutes and Bacteroidetes are the dominant phyla at the phylum level in the cecum of broiler chickens (Zhang Y. et al., 2020), consistent with the results of this experiment. The results of this study indicate that the top four species in terms of total abundance in each group are *Firmicutes, Bacteroidota, Proteobacteria*, and *Desulfobacterota*, among which *Firmicutes* and *Bacteroidota* are dominant phyla. These findings suggest that *Firmicutes* and *Bacteroidota* are dominant microbial communities in the poultry intestine and are less susceptible to external influences. Research has found that *Bacteroidota* can decompose non-fiber carbohydrates and proteins in feed, thereby promoting the development of the gastrointestinal immune system and playing an important role in providing energy and maintaining normal metabolism in animal bodies (Pandit et al., 2018). The higher abundance of *Bacteroidota* is beneficial for the absorption of nutrients in the intestine. This study found that compared with the DQ group, the addition of QG to the diet significantly reduced the relative abundance of Firmicutes and significantly increased the relative abundance of *Bacteroidota*. This is consistent with the improved growth performance of the experimental chickens in this study. The composition and abundance of microbiota in the intestine have a significant impact on the vital activities, immunity, digestive function, and adaptability of poultry, representing important indicators of their health status and production performance (Lynch and Hsiao, 2019). During the growth process of poultry, many factors can lead to an increase in reactive ROS levels in the body, resulting in oxidative stress. Excessive ROS levels can damage the intestinal structure and disrupt the intestinal microbiota, resulting in poultry diseases and impeding their growth and development (Assimakopoulos et al., 2004). Oxidative stress alters the intestinal environment, favoring the proliferation of pathogenic bacteria and the production of harmful metabolites. This, in turn, leads to competition with beneficial microbiota and suppresses their proliferation, further exacerbating the damage caused by oxidative stress to the organism. The results of this study indicate that DQ significantly reduces the abundance of *Lactobacillus* and *Alistipes*, whereas the addition of QG significantly alleviated the decrease in abundance of *Lactobacillus* and *Alistipes*. *Alistipes* is a major producer of short-chain fatty acids such as acetic acid and propionic acid. Acetic acid, propionic acid, and butyric acid are the three most abundant short-chain fatty acids in the intestine, accounting for over 90% of total short-chain fatty acids. These short-chain fatty acids play a regulatory role in energy metabolism, intestinal inflammatory response, and immune function (Li et al., 2017). Parker et al. (2020) found that *Alistipes* may have protective effects against certain diseases, including liver fibrosis, colitis, tumor immunotherapy, and cardiovascular diseases. This further validates that the inclusion of QG in the diet can increase the relative abundance of beneficial microbiota, thereby improving intestinal health.

The Spearman correlation analysis underscores the link between broiler growth performance and cecal microbiota, highlighting the influence of oxidative stress and microbial balance on broiler health. Negative correlations between BW and bacteria such as *Escherichia-Shigella, Subdoligranulum, Romboutsia*, and *Lachnospiraceae_unclassified* suggest their negative impact on growth, potentially through inflammation or nutrient absorption issues. In contrast, the positive correlation between BW and *Alistipes*, a producer of SCFAs, supports its role in enhancing nutrient absorption and energy metabolism. ADFI showed negative correlations with *Escherichia-Shigella, Romboutsia*, and *Lachnospiraceae_unclassified*, while positive correlations with *Erysipelatoclostridium, UCG-005*, and *Bacteroides* indicate their potential role in promoting feed efficiency and gut health. The positive correlation betweenADG and *UCG-005* highlights its beneficial effects on growth. Regarding antioxidant function, *Subdoligranulum* positively correlated with GSH-Px, while SOD correlated positively with *Romboutsia, Lactobacillus*, and *Lachnospiraceae_unclassified*, indicating their role in redox balance. The negative correlations between SOD and *Bacteroides* and *Bilophila* suggests that oxidative stress disrupts beneficial microbes, promoting harmful ones. The addition of QG to the diet mitigated oxidative stress by increasing beneficial bacteria like *Lactobacillus* and *Alistipes*, which produce SCFAs essential for intestinal health and metabolism. QG's ability to restore microbial balance and antioxidant capacity suggests its potential in improving broiler growth and immune response. It is therefore speculated that QG can enhance the antioxidant capacity of broilers by synergistically interacting with intestinal microbiota, reducing oxidative damage caused by oxidative stress, improving the microbial composition related to intestinal health and immune metabolism in the cecum, and ultimately enhancing their growth performance and immune capacity.

## 5 Conclusion

The results of this experiment indicate that the intraperitoneal injection of DQ successfully established a model of oxidative stress in broiler chickens. Supplementing the basal diet with different levels of QG improved the growth performance of broilers, enhanced serum antioxidant capacity, and mitigated oxidative stress damage in the intestine. The mechanism of action of QG may be related to the regulation of the Nrf2/Keap1 signaling pathway. Furthermore, QG supplementation modulated the cecal microbiota. Comprehensive analysis showed that the optimal effect on broiler chickens was achieved with the addition of 20 mg/kg of QG to the diet. This study provides a theoretical foundation for incorporating QG into broiler diets, aiming to enhance their growth performance and antioxidant capabilities.

## Data Availability

The datasets presented in this study can be found in the NCBI repository, accession number PRJNA1173338.
